# Dentition

**Published:** 1885-05

**Authors:** 


### Dentition.

The dangerous period with children is during teething, from six to thirty months. Inflamed gums are quite apt to produce fever, or indigestion, disordered stomach, or convulsions. A little fore-knowledge at such times has saved many a child's life. In case of convulsions, the face and head should be promptly bathed with cold water, and the entire body placed in hot bath. At the same time, with a sharp pocket knife, lance the swolen gum. The attendant feverishness and irritability should be allayed by a soothing aperient medicine. We know of nothing better than Castoria, a well authenticated prescription, the formula of which is widely published. Give the child plenty of air, plenty of exercise and plenty of water.

